# Transcriptome and Coexpression Network Analyses Provide In-Sights into the Molecular Mechanisms of Hydrogen Cyanide Synthesis during Seed Development in Common Vetch (*Vicia sativa* L.)

**DOI:** 10.3390/ijms23042275

**Published:** 2022-02-18

**Authors:** Mingyu Li, Lu Zhao, Qiang Zhou, Longfa Fang, Dong Luo, Wenxian Liu, Iain Robert Searle, Zhipeng Liu

**Affiliations:** 1 State Key Laboratory of Grassland Agro-Ecosystems, Key Laboratory of Grassland Livestock Industry Innovation, Ministry of Agriculture and Rural Affairs, College of Pastoral Agriculture Science and Technology, Lanzhou University, Lanzhou 730020, China; limy19@lzu.edu.cn (M.L.); zhaol19@lzu.edu.cn (L.Z.); zhouq2013@lzu.edu.cn (Q.Z.); fanglf@lzu.edu.cn (L.F.); luod15@lzu.edu.cn (D.L.); liuwx@lzu.edu.cn (W.L.); 2 School of Biological Sciences, The University of Adelaide, Adelaide, SA 5005, Australia; iain.searle@adelaide.edu.au

**Keywords:** common vetch, seed development, HCN, transcriptome

## Abstract

The common vetch (*Vicia sativa* L.) seed is an ideal plant-based protein food for humans, but its edible value is mainly limited by the presence of cyanogenic glycosides that hydrolyze to produce toxic hydrogen cyanide (HCN), and the genes that regulate HCN synthesis in common vetch are unknown. In this study, seeds from common vetch at 5, 10, 15, 20, 25, 30, and 35 days after anthesis were sampled, and the seven stages were further divided into five developmental stages, S1, S2, S3, S4, and S5, based on morphological and transcriptome analyses. A total of 16,403 differentially expressed genes were identified in the five developmental stages. The HCN contents of seeds in these five stages were determined by alkaline titration, and weighted gene coexpression network analysis was used to explain the molecular regulatory mechanism of HCN synthesis in common vetch seeds. Eighteen key regulatory genes for HCN synthesis were identified, including the *VsGT2*, *VsGT17* and *CYP71A* genes, as well as the *VsGT1* gene family. *VsGT1*, *VsGT2*, *VsGT17* and *CYP71A* jointly promoted HCN synthesis, from 5 to 25 days after anthesis, with *VsGT1-1*, *VsGT1-4*, *VsGT1-11* and *VsGT1-14* playing major roles. The HCN synthesis was mainly regulated by *VsGT1,* from 25 to 35 days after anthesis. As the expression level of *VsGT1* decreased, the HCN content no longer increased. In-depth elucidation of seed HCN synthesis lays the foundations for breeding common vetch with low HCN content.

## 1. Introduction

The global demand for protein is predicted to increase by 50% by 2050 [1,2]. As the global population increases, the demand for protein from animal sources has grown. With the expansion of livestock farming, overgrazing has contributed to a reduction in terrestrial biodiversity and increased greenhouse gas emissions, climate change and global warming [1]. To meet the increasing protein demand and to protect the ecosystem, more sustainable protein foods are needed to partially replace animal sources of protein. Inexpensive plant proteins, such as legume seeds, can address the shortage of protein supply and are environmentally friendly, making them ideal for developing countries with rapidly growing populations [3].

Common vetch (*Vicia sativa* L.), a self-pollinated annual legume, with a genome size of 1.5 Gb, is drought- and cold-tolerant and is widely grown as food for humans and livestock and used extensively for soil improvement. Common vetch is one of the most genetically and phenotypically variable species of *Vicia*, with great variability in plant morphology, maturity, stem length and thickness, leaf width, pod setting, pod and seed size, and nutritional indicators [4,5,6]. It is certainly an attractive source of plant-based protein for many regions of the world, especially for dryland agriculture [7]. Common vetch has been found to have been food for early humans in archaeological investigations of Neolithic and Bronze Age sites, as well as ancient Roman settlements in Europe. During the famine years, people in certain regions of Europe and the Middle East detoxified common vetch for consumption in brine [8]. Common vetch contains a high concentration of crude protein in the seeds, ranging from 24% to 32% [2]. This concentration is comparable to those in lentils (*Lens culinaris* M.) and chickpeas (*Cicer arietinum* L.) [9]. Common vetch seeds contain 18 amino acids, with an essential amino acid/nonessential amino acid ratio of approximately 0.7, which is much higher than the 0.38 recommended by the World Health Organization [10]. In addition to its nutritional value, common vetch contains a large number of chemical components with pharmacological activity [8,11]. However, common vetch cannot be fed in large quantities to monogastric animals and is currently used mainly as a feed for ruminants. It can be argued that a higher value return can be observed by using it as a food crop for humans [2]. The market circulation of common vetch seeds as edible legumes is hampered by a series of antinutritional factors. Chief among these factors is a class of cyanogenic glycosides, named vicianin, which hydrolyze to produce hydrogen cyanide (HCN) [12].

As a secondary metabolite with defense functions, cyanogenic glycosides have been found in more than 3000 higher plants, including ferns, gymnosperms and angiosperms [13,14]. Cyanogenic glycosides are stored in vacuoles. When herbivores or pathogens damage cyanogenic plant tissues, β-glucosidase and α-hydroxynitrile lyase degrade cyanogenic glycosides in cells, generate and release toxic HCN, glucose, aldehydes or ketones, and produce chemical defense reactions [15]. Cyanogenic glycosides have many structural phenotypes in different plants, such as amygdalin, dhurrin [16], linamarin and lotaustralin [17], and vicianin [12]. The biosynthesis of cyanogenic glycosides in plants involves three kinds of enzymes, including two cytochrome P450 genes, belonging to the *CYP79* and *CYP71* families, and a glucose transferase, UGT85B1 [18].

However, HCN synthesis in common vetch has rarely been reported. Moreover, which genes are involved in the synthesis of HCN in common vetch is unknown. Therefore, in this study, common vetch seeds at seven different developmental stages were sampled and combined with transcriptomic data, HCN assay and weighted gene coexpression network analysis (WGCNA) to establish a coexpression network of HCN synthesis genes. The main regulatory genes of HCN synthesis during common vetch seed development were identified. The molecular regulatory mechanism of HCN synthesis in common vetch seeds was elucidated, which could provide a theoretical basis for breeding new common vetch cultivars with low HCN content.

## 2. Results and Discussion

### 2.1. Morphological and Physiological Changes during Seed Development

**Figure 1:**
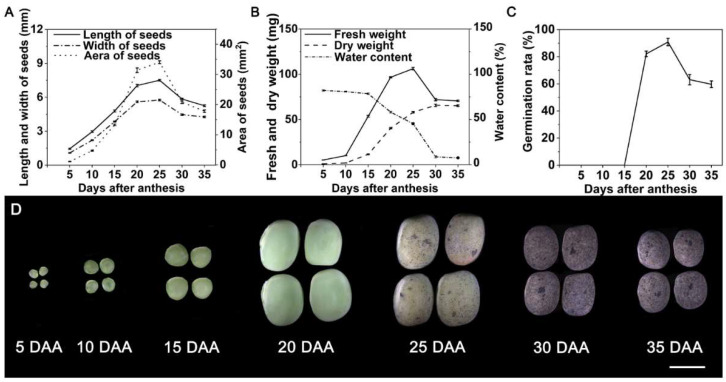
Morphology changes during seed development. (**A**) Changes in length, width and area of seeds. (**B**) Changes in fresh weight, dry weight and water content of seeds. (**C**) Changes in the germination rate of seeds. (**D**) Morphological changes at different developmental stages from 5 DAA to 35 DAA in the seeds of common vetch. Scale bar = 5 mm.

Seven developmental stages of common vetch seeds, from generation to maturity, were selected to study the gene expression patterns during seed development. From 5 days to 25 days after anthesis (DAA), seed size (length, width and area) and weight gradually increased, and seed water content gradually decreased, especially during the period of 15 to 25 DAA. The seed size and weight gradually decreased and tended to be stable as the seed matured after 25 DAA (Figure 1A,B,D). The seed began to have viability after 15 DAA, and its germination rate increased first and then decreased (Figure 1C). The germination rate was the highest at 25 DAA, decreased gradually, and became stable after 30 DAA, indicating that hard seeds appeared with the development and maturity of the seed coat (Figure 1C) between 25 and 35 DAA. During the period of 5 to 15 DAA, the seeds were dark green, changed to light green at 20 DAA, and turned to light brown from 25 DAA, and black spots of different sizes appeared on the surface. After 30 DAA, the seeds turned dark brown and were unchanged in later stages (Figure 1D).

Seed size and weight gradually increase as the cotyledons emerge and fill all areas of the seed coat, prior to seed maturation. With seed maturity, water loss in the seed results in decreases in seed size and weight until the seed is fully mature [19,20,21]. In the early stage of seed development, the germination rate gradually increases as the seed embryo is well developed. Then, the germination rate decreases after the seeds begin to mature. It has been reported that the germination rate is negatively correlated with lignin content, probably because the lignin-rich seed coat prevents the seed from absorbing water and nutrients [22,23]. In this study, the germination rate of common vetch seeds was the highest at 25 DAA, suggesting that harvesting seeds at this stage may reduce the hardness rate of common vetch seeds.

### 2.2. Illumina Sequencing, Assembly and Functional Annotation

RNA-Seq was carried out with RNA, from seven stages of seed development (5 to 35 DAA), to gain an overall view of seed development at the transcriptional level. Analyses were performed with three biological replications at each stage. After removing low-quality data from each library, 137.63 Gb of clean data were generated (Table S1). The Q30 of the raw data has a quality rating of 93.89% to 95.75%, suggesting high-quality reads that are worth investigating further. Since common vetch had no available reference genome, 144,277 transcripts and 44,934 unigenes were assembled by de novo assembly of all 458,744,514 reads (Table S2). Eight candidate unigenes were randomly selected for qRT–PCR validation, to verify the accuracy and repeatability of the transcriptome analysis in the present study (Table S3). The FPKM values were significantly correlated with the expression levels of these unigenes (Figure S1), indicating that the transcript data generally matched the qRT–PCR results in the current study.

**Figure 2:**
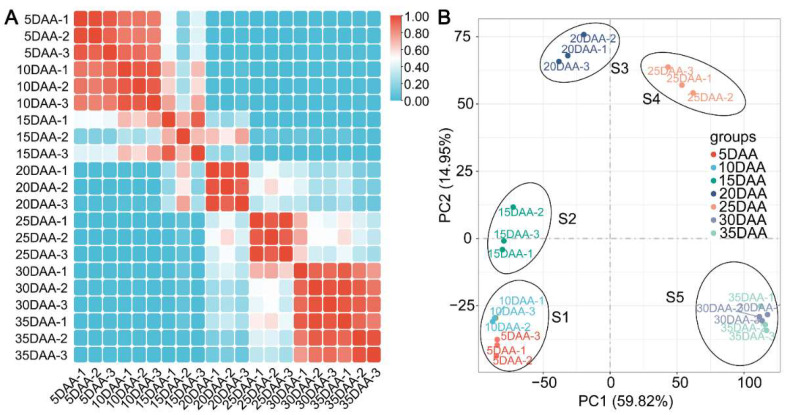
(**A**) Correlation analysis and (**B**) principal component analysis (PCA) of all samples during seed development. The seven stages were classified into five developmental stages, S1 (5–10 DAA), S2 (15 DAA), S3 (20 DAA), S4 (25 DAA) and S5 (30–35 DAA), based on correlation and principal component analyses.

Pearson’s correlation coefficient was used to test the biological reproducibility of this study, which showed that there was a high correlation between samples taken from the same biological replicates, with values ranging from 0.812–0.927. However, the correlations were different between the samples, with values ranging from 0.190 to 0.896, except at 5 DAA and 10 DAA, 30 DAA and 35 DAA (Figure 2A). All samples collected during seed development were subjected to principal component analysis (PCA). Consistent with the correlation analysis, samples from different biological replicates were clustered separately, with 5 DAA and 10 DAA, 30 DAA and 35 DAA clustered together. The seven stages were classified into five developmental stages, S1 (5–10 DAA), S2 (15 DAA), S3 (20 DAA), S4 (25 DAA) and S5 (30–35 DAA), based on correlation and principal component analyses (Figure 2B).

The 44,934 unigenes were annotated by performing BLASTX searches against the sequences in the NR, SwissProt, GO, COG, and KEGG databases. As a consequence, 33,433 unigenes (74.40% of all unigenes) were assigned one or more putative functions from these databases (Figure S2A, Table S4). A total of 28,696 unigenes were annotated from the NR database. Further analysis of the matched sequences revealed that the common vetch transcripts were similar to those of *Medicago sativa* (34.7%), *Trifolium pretense* (16.6%), *Trifolium subterranean* (14.6%) and *Cicer arietinum* (13.1%) (Figure S2B). Within biological process classifications, unigenes were involved in cellular processes, metabolic processes, and biological regulation. In biological process classifications, unigenes were associated with cellular processes and metabolic processes. Cellular anatomical entities and intracellular components constituted the most common classifications of cellular components. In the classification of molecular functions, binding and catalysis were predominant (Figure S3). The key GO terms corresponded to the fact that cells divide frequently during seed development.

Because of the lack of sequencing data for *Vicia* species, the number of annotated unigenes in all databases (NR (National Center for Biotechnology Information, MD, USA), SwissProt (Expasy, Swiss Institute of Bioinformatics, Lausanne, Switzerland), GO (Golang, Orlando, FL, USA), COG (National Library of Medicine, MD, USA), and KEGG (Kanehisa Laboratories, Tokyo, Japan)) accounts for 74.4% of the total number of unigenes, which should be sufficient. In previous studies, in other species, such as *Dendrocalamus latiflorus* and *Gardenia jasminoides*, annotated single genes accounted for 55% and 69% of the total number of unigenes, respectively [24,25]. Functional classification of common vetch genes, based on GO annotations, identified genes related to ‘biological processes’, ‘cellular components’ and ‘molecular functions’. Genes identified as ‘biological processes’ during seed development in common vetch were also identified in *Glycine max*, *Fagopyrum* *tararicum* and *Artemisia sphaerocephala* [20,26,27], indicating that these genes have significant metabolic activity during seed development.

### 2.3. Differentially Expressed Genes during Seed Development

**Figure 3:**
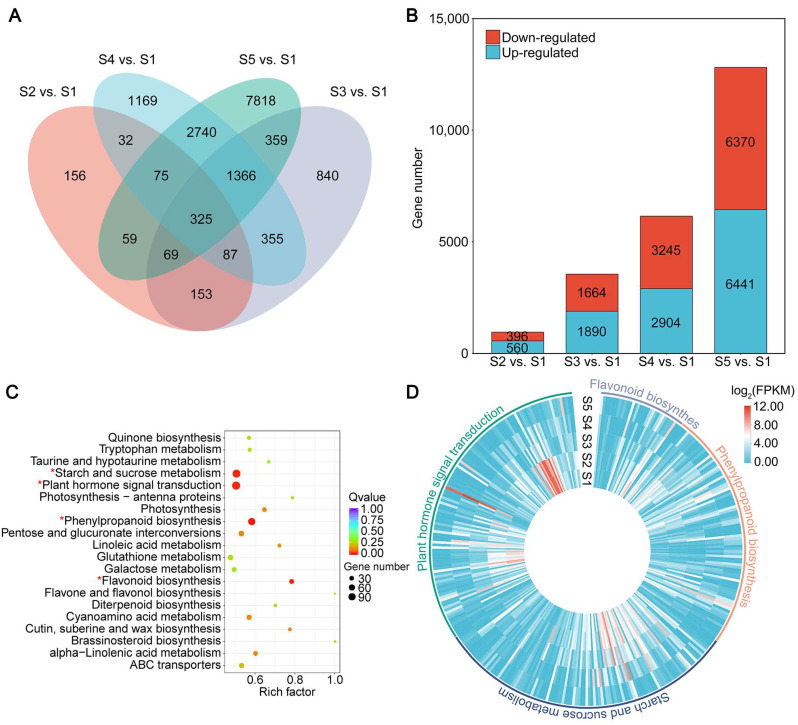
Summary of differentially expressed genes (DEGs) identified in the common vetch seed transcriptomes. (**A**) Venn diagrams of DEGs detected in S2 vs. S1, S3 vs. S1, S4 vs. S1, and S5 vs. S1. (**B**) Numbers of DEGs identified in S2 vs. S1, S3 vs. S1, S4 vs. S1, and S5 vs. S1. (**C**) KEGG enrichment analysis with the DEGs during seed development. The * represents significantly enriched pathways. (**D**) Gene expression patterns of the four pathways significantly enriched in DEGs of common vetch seed.

There were 16,403 DEGs identified by DESeq, based on FPKM values, of which 956, 3554, 6149 and 12,811 DEGs were identified in S2 vs. S1, S3 vs. S1, S4 vs. S1 and S5 vs. S1; 560, 1890, 2940 and 6441 upregulated, and 396, 1664, 3245, and 6370 downregulated, respectively (Figure 3A,B). The results indicated that the expression levels of genes differed significantly between stages of development in common vetch seeds. More than 10,000 unigenes were assembled, and thousands of DEGs were identified in the current study, which is consistent with the results of previous studies with different varieties or tissues of common vetch under different treatments [28,29].

KEGG pathways were identified to comprehensively understand the metabolic pathways involved in DEGs (Table S5). A total of 16,403 DEGs were annotated into 120 pathways, of which the following four pathways were significantly enriched (*q*-value < 0.05): plant hormone signal transduction, starch and sucrose metabolism, phenylpropanoid biosynthesis, and flavonoid biosynthesis (Figure 3C).

Plant hormones are important signals that control seed development, maturation and nutrient accumulation [30]. Previous studies have shown that plant hormones affect seed size and shape, [31,32,33] auxin biosynthesis and signaling, [34,35] starch biosynthesis, seed coat development, [36,37] seed yield, and embryo growth [38]. Starch is the major form of carbohydrate accumulation during seed development and is the main nutrient that enables seed expansion [26]. Genes in the starch biosynthesis pathway, including *SUS, UGPase*, *AGPase*, *GBSS*, *SS*, *BE*, and *DBE*, have been fully characterized [39]. Phenylpropanoids are the largest component of plant secondary metabolites during seed development [40,41]. Flavonoids are one of the most widely studied nutrients in seeds, and common vetch is rich in flavonoids [8,26]. In this study, the four significantly enriched pathways of plant hormone signal transduction, starch and sucrose metabolism, phenylpropanoid biosynthesis, and flavonoid biosynthesis included 118, 112, 88, and 32 DEGs, respectively (Figure 3D). Similar pathways have been previously reported to be enriched, suggesting that genes in these pathways play important roles in seed development [26,30,41].

### 2.4. HCN Content in Seed and WGCNA of HCN Synthesis Genes

**Figure 4:**
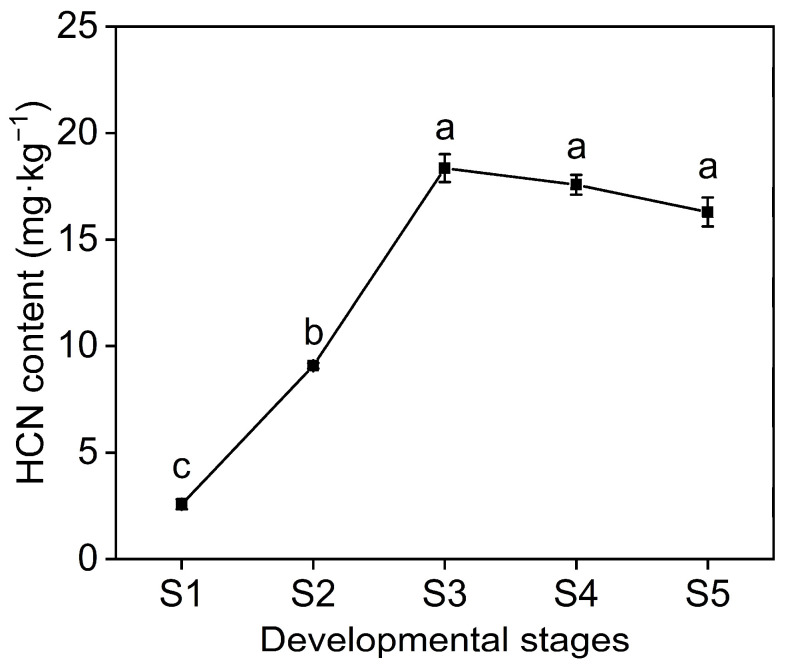
HCN contents in common vetch seeds at different developmental stages. Different letters “a, b, c” indicate significant differences at *p* < 0.05. S1 to S5 indicate the five developmental stages.

As the seeds developed, the HCN content was observed to increase rapidly, from 2.58 mg/kg in S1 to 18.36 mg/kg in S3, which was the highest, with an increase of 85.95% from S1 to S3. It then decreased slightly and reached 16.30 mg/kg at S5 (Figure 4), which is higher than the safe level of 10 mg/kg, set by the World Health Organization (Geneva, Switzerland) for cyanide content in cassava foods [42].

**Figure 5:**
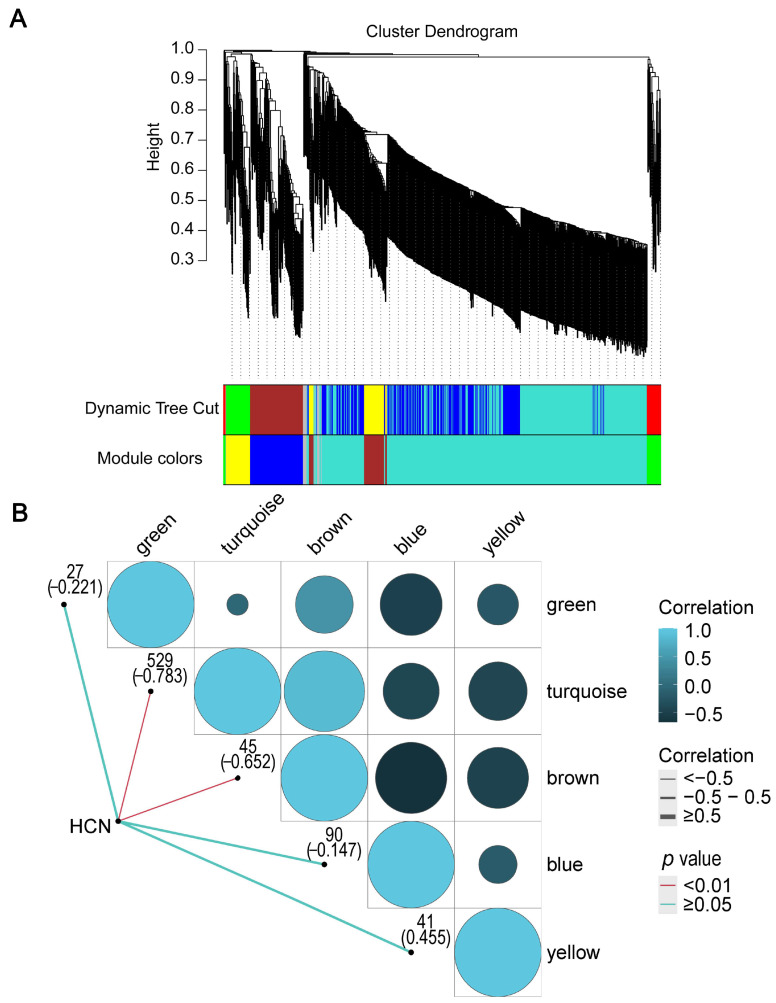
(**A**) Weighted gene coexpression network analysis (WGCNA) of HCN-forming genes. (**B**) Correlation of modules and sample.

Among the DEGs of common vetch seeds at different developmental stages, a total of 745 HCN synthesis-related genes were identified (Table S6). To construct a weighted gene coexpression network consisting of five modules, named green, turquoise, brown, blue and yellow, WGCNA was used (Figure 5A). There were 27, 529, 45, 90, and 41 genes in the green, turquoise, brown, blue and yellow modules, respectively. Another 13 genes in the gray module were excluded (Figure 5B). Further analysis revealed that the correlation coefficients between the modules and HCN content ranged from -0.783 to 0.455. In the five modules, the turquoise module had the highest correlation coefficient with HCN content and was significantly correlated (*p* < 0.001), suggesting that the turquoise module plays a major role in HCN synthesis (Figure 5B). A correlation analysis between the module membership (MM) and gene significance (GS) of the five modules was performed, in which a significant correlation between MM and GS of the turquoise module was found (*R* = −0.940, *p* = 3.13 × 10^−257^) (Figure S4). In the turquoise module, genes with high MM tended to have high GS, and GS and MM exhibited a very significant correlation, implying that genes of the turquoise module also tend to be highly correlated with HCN content. The above results indicated that the turquoise module played a critical role in the gene network of HCN synthesis. The high correlation between the brown and turquoise modules suggests that some genes in the brown module may also be involved in the synthesis of HCN. Furthermore, WGCNA was used to search genes with similar expression patterns to identify genes that may be involved in biological processes. In a previous study, the coexpression network of seed mucilage-forming genes of *Artemisia sphaerocephala* was successfully constructed using WGCNA, and a key module was identified [43].

### 2.5. Key Regulatory Pathways for HCN Synthesis

The KEGG pathway analysis of key module genes showed that the top ten pathways with gene numbers were phenylpropanoid biosynthesis, starch and sucrose metabolism, cyanoamino acid metabolism, flavonoid biosynthesis, brassinosteroid biosynthesis, flavone and flavonol biosynthesis, isoflavonoid biosynthesis, cutin, suberine and wax biosynthesis, zeatin biosynthesis, and stilbenoid, diarylheptanoid and gingerol biosynthesis, whose gene numbers were twenty, nineteen, twelve, six, five, four, three, three, two and two, respectively (Table 1). Among the top ten KEGG pathways of key genes, flavonoid biosynthesis, flavone and flavonol biosynthesis, isoflavonoid biosynthesis, and stilbenoid, diarylheptanoid and gingerol biosynthesis belonged to secondary metabolite synthesis. Starch and sucrose metabolism belonged to carbohydrate metabolism, and cyanoamino acid metabolism belonged to amino acid metabolism. The above results were consistent with the fact that cyanogenic glycosides are naturally occurring plant secondary metabolites, derived from amino acids, which consist of a sugar moiety with an α-hydroxynitrile-type aglycone attached to it [44,45]. Plant hormones are important factors in regulating secondary metabolite biosynthesis, and the application of jasmonic acid has been reported to enhance cyanogenesis in lima bean (*Phaseolus lunatus* L.) by inducing the expression of biosynthetic pathway genes [46]. In this study, hormone biosynthesis (brassinosteroid biosynthesis and zeatin biosynthesis) was another key regulatory pathway affecting HCN synthesis, suggesting that hormones can also regulate HCN synthesis in common vetch. The specific mechanism of hormone regulation of HCN synthesis needs to be further investigated. Phenylpropanoids contribute to plant responses to biotic and abiotic stimuli, and they are not only indicators of plant stress responses under different treatments but also key mediators of plant resistance to pests [40]. The presence of the phenylpropanoid biosynthesis pathway in the current study confirmed that the nature of HCN synthesis in common seeds was a chemical defense.

**Figure 6:**
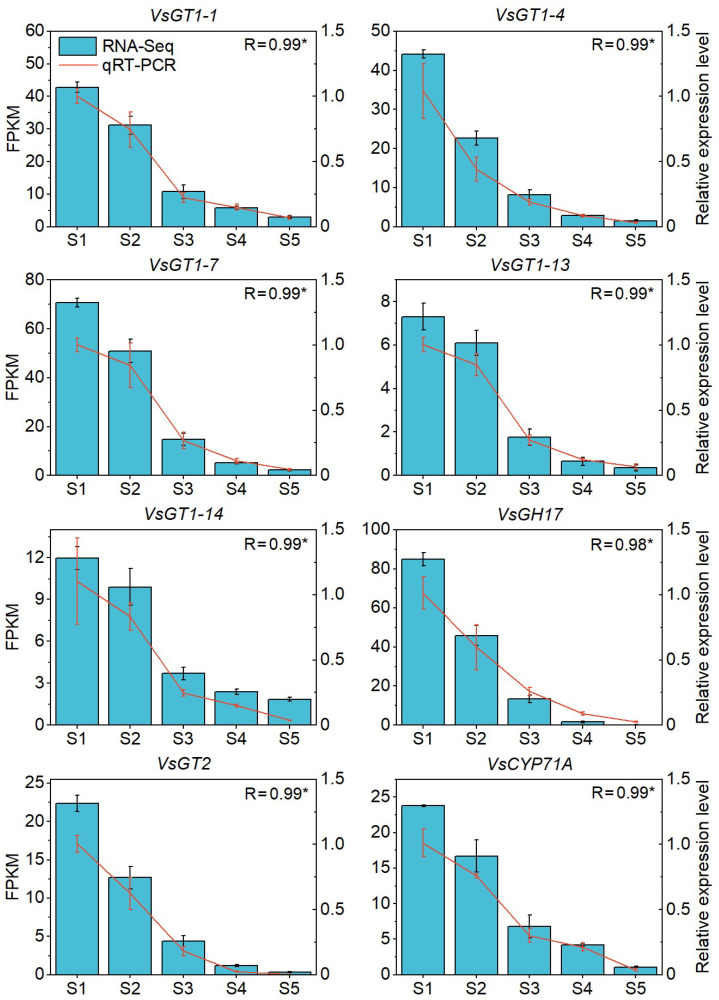
The qRT-PCT confirmation of eight of the key regulatory genes in HCN synthesis. The Y-axis on the left side of each chart indicates the expression level (FPKM) of RNA-seq. The Y-axis on the right side of each chart indicates the relative expression of qRT–PCR, * *p* < 0.05.

Genes with a greater GS and intramodular connectivity (kIN) play a more significant role in modules [47]. In the present study, eighteen key regulatory genes were identified from the key modules, using GS < −0.7 and kIN > 280 as criteria (Table S7). These eighteen key regulatory genes were from four gene families, including *glycosyltransferase family 1 (GT1)*, *glycosyltransferase family 2 (GT2)*, *glycoside hydrolase family 17 (GT17)* and *CYP71A*, and were designated *VsGT1-1* to *VsGT1-15*, *VsGT2*, *VsGT17* and *VsCYP71A*. In addition, the expression patterns of eight of the key regulatory genes were selected and validated by qRT–PCR (Table S3, Figure 6).

**Figure 7:**
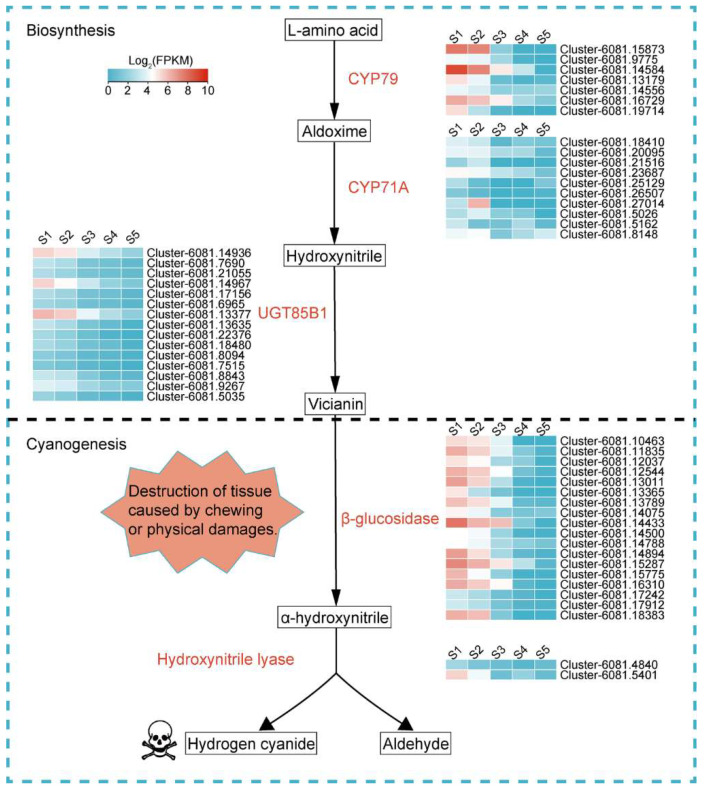
Synthesis pathway and regulation of HCN in common vetch. Above the black dashed line is the process of vicianin biosynthesis, and below the black dashed line is the process of vicianin cyanogenesis to produce HCN. *CYP79*, *CYP71A*: Cytochrome P450 enzymes; UGT85B1: UDP-glycosyltransferase.

**Figure 8:**
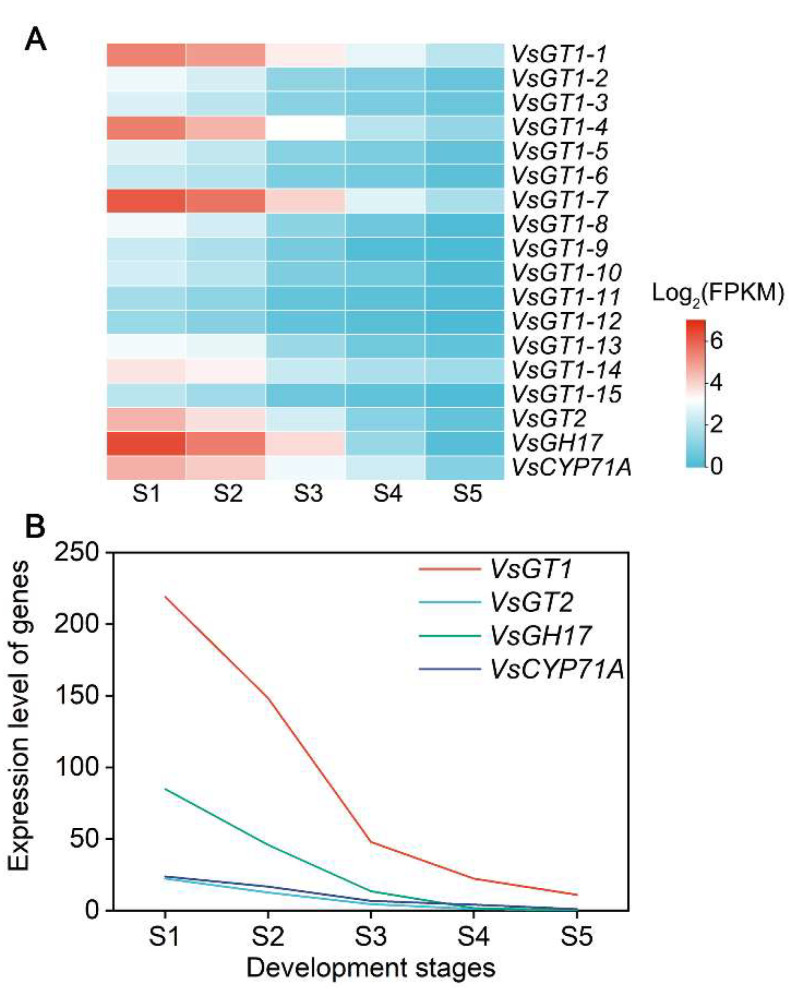
The expression levels of (**A**) key regulatory genes and (**B**) key regulatory gene families.

During hydrocyanic acid synthesis, the transformation of amino acids to aldoxime is catalyzed by the P450 enzyme *CYP79*, and another P450 enzyme *CYP71A* is responsible for the subsequent conversion of aldoxime to hydroxynitrile. Hydroxynitrile is then glycosylated and stabilized by UDP-glycosyltransferase (UGT85B1), resulting in the formation of vicianin. When herbivores or pathogens damage plant tissues, β-Glucosidase in the tissue meets and degrades vicianin, followed by α-Hydroxynitrile lyase, which degrades intracellular vicianin, generating and releasing toxic HCN, as well as aldehyde, i.e., a chemical defense reaction (Figure 7). The expression trends of genes related to HCN synthesis were consistent and trended downward (Figure 8A). Among the fifteen *VsGT1* genes, *VsGT1-1*, *VsGT1-4*, *VsGT1-7* and *VsGT1-14* were expressed at higher FPKM values and decreased from 42.78, 44.18, 70.74 and 11.96 to 3.03, 1.49, 2.28 and 1.86 from S1 to S5, respectively. Overall, *VsGT1-1* and *VsGT1-7* were expressed at higher levels in S1–S3 and rapidly decreased from S3 to S5, and the remaining thirteen *VsGT1* genes were expressed at higher levels from S1 to S2 and rapidly decreased from S2 to S5. *VsGT2*, *VsGH17* and *VsCYP71A* decreased from 22.37, 84.89 and 23.79 in S1 to 0.33, 0.16 and 1.06 in S5, respectively. The expression levels of key regulatory gene families were analyzed (Figure 8A). The *VsGT1* gene family had the highest expression levels from S1 to S3, with 219.05, 148.48 and 47.94, respectively, which was consistent with the rapid synthesis of HCN in S1–S3 (Figure 4 and Figure 8B). The expression levels of *VsGT1*, *VsGT2*, *VsGH17* and *VsCYP71A* all decreased continuously in S3–S5, with the expression levels of *VsGT2*, *VsGH17* and *VsCYP71A* decreasing more rapidly (Figure 8B). The above results indicated that *VsGT1*, *VsGT2*, *VsGT17* and *CYP71A* together, promoted HCN synthesis in S1–S3, with *VsGT1-1*, *VsGT1-4*, *VsGT1-11* and *VsGT1-14* playing major roles. HCN synthesis was mainly regulated by *VsGT1* from S3 to S5. As the expression level of *VsGT1* decreased, the HCN content no longer increased.

## 3. Materials and Methods

### 3.1. Plant Materials

Healthy and plump seeds of the common vetch cultivar ‘Lanjian No. 3’ were provided by Lanzhou University (Lanzhou, Gansu, China). All the seeds were sown in the experimental field of Lanzhou University, at an elevation of 1520 m, with an average annual precipitation of 350 mm and an average annual temperature of 6.7 °C. The plants were single planted in a plot, and the row spacing and plant spacing were 50 cm. The flowers in full bloom with dorsal petal fully expanded were marked, and then seeds were harvested every five days until 35 days [28]. Finally, seeds at seven different developmental stages (5–35 DAA) were collected. The seeds collected from three plants were mixed as a single sample with three biological replications in each developmental stage. A total of 21 samples were collected and immediately frozen with liquid nitrogen and then stored at −80 °C for total RNA extraction.

### 3.2. Morphological and Physiological Characteristics of Seeds

Fifty seeds were randomly harvested from three plants for the determination of seed length, width and area, and repeated three times. The seed length, width and area were measured by VideometerLab 4 (Videometer, Horsholm, Denmark).

The seeds were harvested using the same method as above for the determination of fresh weight, dry weight and germination rate. The fresh weight of each seed was measured immediately after the seeds were harvested. Then, the seeds were dried at 60 °C to measure the dry weight of each seed, and the water content was calculated by water content (%) = (fresh weight-dry weight)/fresh weight × 100. The dried seeds were sterilized with 1.0% (*v*/*v*) sodium hypochlorite for five minutes and then washed with distilled water three times. The seeds were placed on double-layer sterile filter paper soaked in distilled water and placed in a 9-cm Petri dish. Then, the cells were germinated at 20 °C and supplemented with distilled water every 24 h. The radicle protruding from the seed coat was regarded as germination, and seeds that did not become imbibited after 14 days were counted as hard seeds.

### 3.3. HCN Content Determination

The HCN content in common vetch seeds was determined by alkaline titration. Twenty grams of air-dried common vetch seeds were ground with a blender and transferred to a Kjeldahl flask, and 200 mL water was mixed with the sample. The solution was distilled after two hours. A flask containing 20 mL of 2.5% NaOH solution was used to collect the distillation until it reached a definite volume. After adding 8 mL of 6 M NH_4_OH and 2 mL of 5% KI solution into the distillation, 0.02 M AgNO_3_ was used for titration with a microburette. The HCN content was calculated as follows: HCN content (mg/kg) = mL of 0.02 M AgNO_3_ × 50/1.08 [48].

### 3.4. RNA Sequencing and Transcriptome Analysis

Total RNA was isolated using TRIzol reagent (Invitrogen, Carlsbad, CA, USA) according to the manufacturer’s instructions. Agarose gel electrophoresis and a NanoPhotometer spectrophotometer (IMPLEN, Westlake Village, CA, USA) were used to determine the concentration, quality and integrity of the isolated RNA. RNA samples meeting the requirements (total RNA ≥ 1.0 μg, RNA concentration ≥ 50 ng/μL) were sent to Novogene Co. Ltd. (Tianjin, China) for transcriptome sequencing on the Illumina HiSeq platform. Quality control analysis of the raw reads was first performed with FastQC (Babraham Institute, Cambridge, UK). Reads containing adapters, reads containing poly-N and low-quality reads were removed from the raw data to obtain clean data (clean reads). The Q20, Q30, GC content and sequence duplication level were also calculated for the clean data. All downstream analyses were based on clean data with high quality. All 21 libraries (5–35 DAA, three replicates per treatment) were merged into one library. De novo assembly of the common vetch seed transcriptome was generated using Trinity software. All assembled unigenes were annotated in the Nr (NCBI nonredundant protein sequences), Nt (NCBI nonredundant nucleotide sequences), Pfam (Protein family, European Molecular Biology Laboratory, Barcelona, Spain), KOG (Health Sciences Communities, Pittsburgh, PA, USA)/COG (Clusters of Orthologous Groups of proteins), Swiss-Prot (a manually annotated and reviewed protein sequence database), KO (KEGG Ortholog), and GO (Gene Ontology) databases.

Fragments per kilobase per million (FPKM) were used to estimate gene expression levels. Principal component analysis of samples was performed on regularized logarithmic reads. The DEseq package was used to identify differentially expressed genes (DEGs). Unigenes with adjusted *p*-values < 0.05 and absolute value of log_2_ fold change ≥2 were designated DEGs. ClusterProfiler was employed to perform GO and KEGG enrichment analyses to infer the putative functions of DEGs.

### 3.5. Establishment of the Gene Coexpression Network

The R package (The R Foundation, Vienna, Austria) WGCNA V1.41-1 was used to perform the WGCNA. An applicable soft-threshold power based on the scale-free topology criterion was used to transform the correlation matrix between all genes into a signed weighted adjacency matrix for calculating the topological overlap matrix (TOM). All genes were then clustered using dynamic tree cuts to form modules with a minimum module size of 20 genes.

### 3.6. qRT–PCR Analysis

Transcript-specific primers were designed with Primer 6 (Table S3) and synthesized by Tsingke Biotechnology (Xi’an, China) in this study. Following this, qRT–PCR was performed using 2xSG Fast qPCR Master Mix (Sangon Biotech, Shanghai, China) on a CFX96 Touch™ Real-Time PCR Detection System (Bio-Rad, Hercules, CA, USA) under the following parameters: 95 °C for 30 s, 39 cycles of 95 °C for 5 s and 60 °C for 30 s. The common vetch actin gene (*Unigene68614*) was selected as an internal standard to calculate relative expression levels by the 2^−^^ΔΔ^^Ct^ method [28].

### 3.7. Statistical Analysis

Duncan’s multiple test (*p* < 0.05) was performed using SPSS software (version 21.0, IBM, Amonk, NY, USA). Data were obtained from at least three independent biological replicates. Heatmaps were generated using TBtools software [49].

## 4. Conclusions

In conclusion, eighteen key genes for HCN synthesis were identified, and the molecular mechanism of HCN synthesis in common vetch seeds was proposed in this study. During the development of common vetch, *VsGT1*, *VsGT2*, *VsGT17* and *CYP71A* jointly promoted HCN synthesis, from 5 to 25 days after anthesis, with *VsGT1-1*, *VsGT1-4*, *VsGT1-11* and *VsGT1-14* playing major roles. We found that HCN synthesis was mainly regulated by *VsGT1* from 25 to 35 days after anthesis. As the expression level of *VsGT1* decreased, the HCN content no longer increased. This study provides a theoretical basis for the cultivation of common vetch with low HCN content and provides a new solution to the growing protein demand.

## Figures and Tables

**Table 1:** Top ten pathways of HCN synthesis genes.

Pathway	Ko Pathway	Gene Number
Phenylpropanoid biosynthesis	ko00940	20
Starch and sucrose metabolism	ko00500	19
Cyanoamino acid metabolism	ko00460	12
Flavonoid biosynthesis	ko00941	6
Brassinosteroid biosynthesis	ko00905	5
Flavone and flavonol biosynthesis	ko00944	4
Isoflavonoid biosynthesis	ko00943	3
Cutin, suberine and wax biosynthesis	ko00073	3
Zeatin biosynthesis	ko00908	2
Stilbenoid, diarylheptanoid and gingerol biosynthesis	ko00945	2

## Data Availability

The sequence data reported in this paper have been submitted to the NCBI sequence read archive (BioProject accession: PRJNA778324).

## Supplementary Materials

The following are available online at: https://www.mdpi.com/article/10.3390/ijms23042275/s1.
