# Supplementary material for: Transcriptome and Coexpression Network Analyses Provide In-Sights into the Molecular Mechanisms of Hydrogen Cyanide Synthesis during Seed Development in Common Vetch (Vicia sativa L.)
Source: Int J Mol Sci. 2022 Feb 18;23(4):2275. doi: 10.3390/ijms23042275 (PMC8878228; doi:10.3390/ijms23042275)
Supplement: Supplementary file 1 [file ijms-23-02275-s001.zip › ijms-1565579-supplementary materials figures.pdf]

International Journal of
Molecular Sciences

Supplementary materials

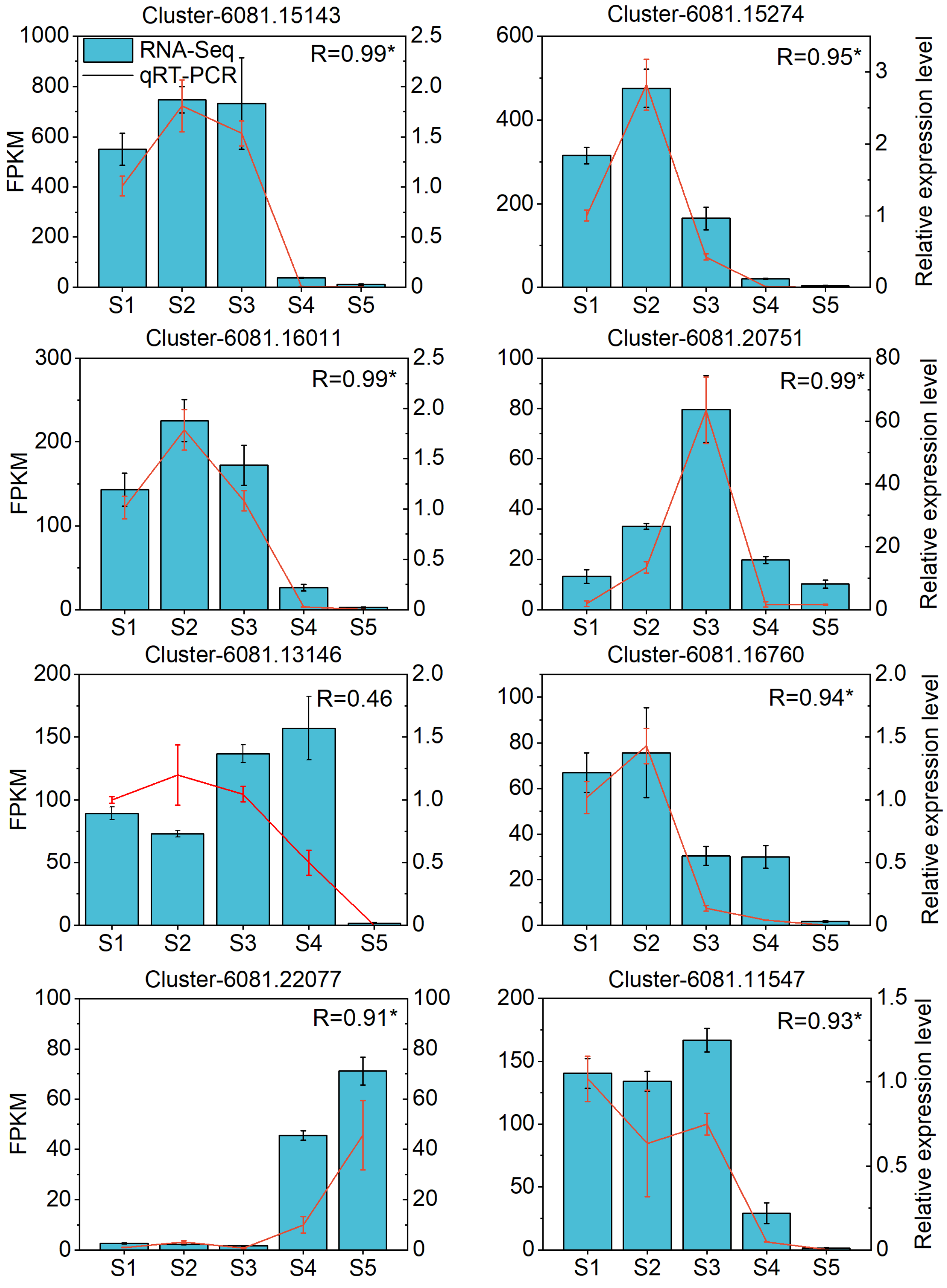

Figure S1. qRT–PCR validation of eight randomly selected genes. The Y-axis on the left side of each chart indicates the expression level (FPKM) of RNA-seq. The Y-axis on the right side of each chart indicates the relative expression of qRT–PCR.

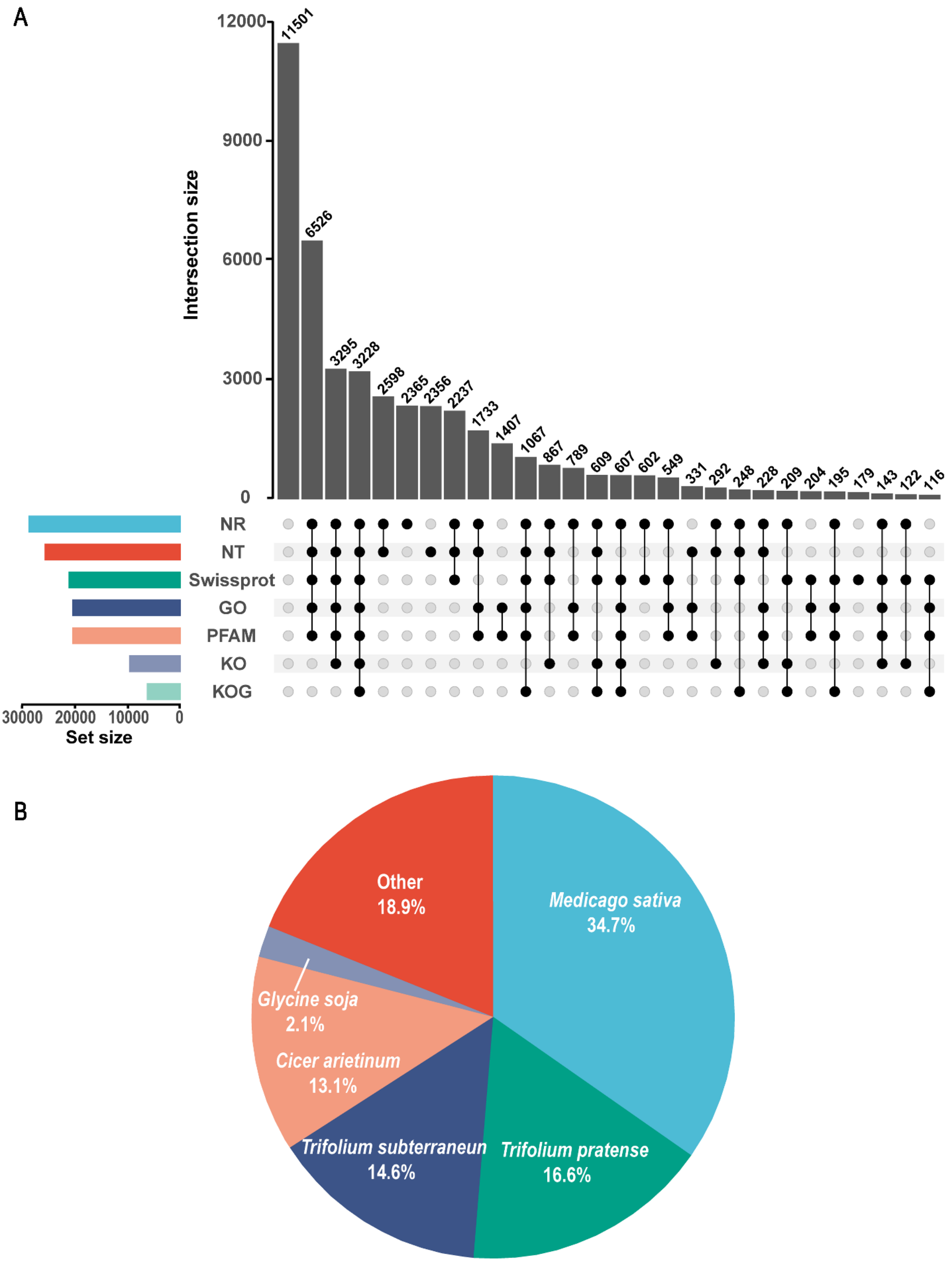

Figure S2. Statistics of transcripts annotation. (**A**) Number of transcripts annotated to the seven databases. (**B**) The top five species distribution of transcripts that were annotated on the basis of homology.

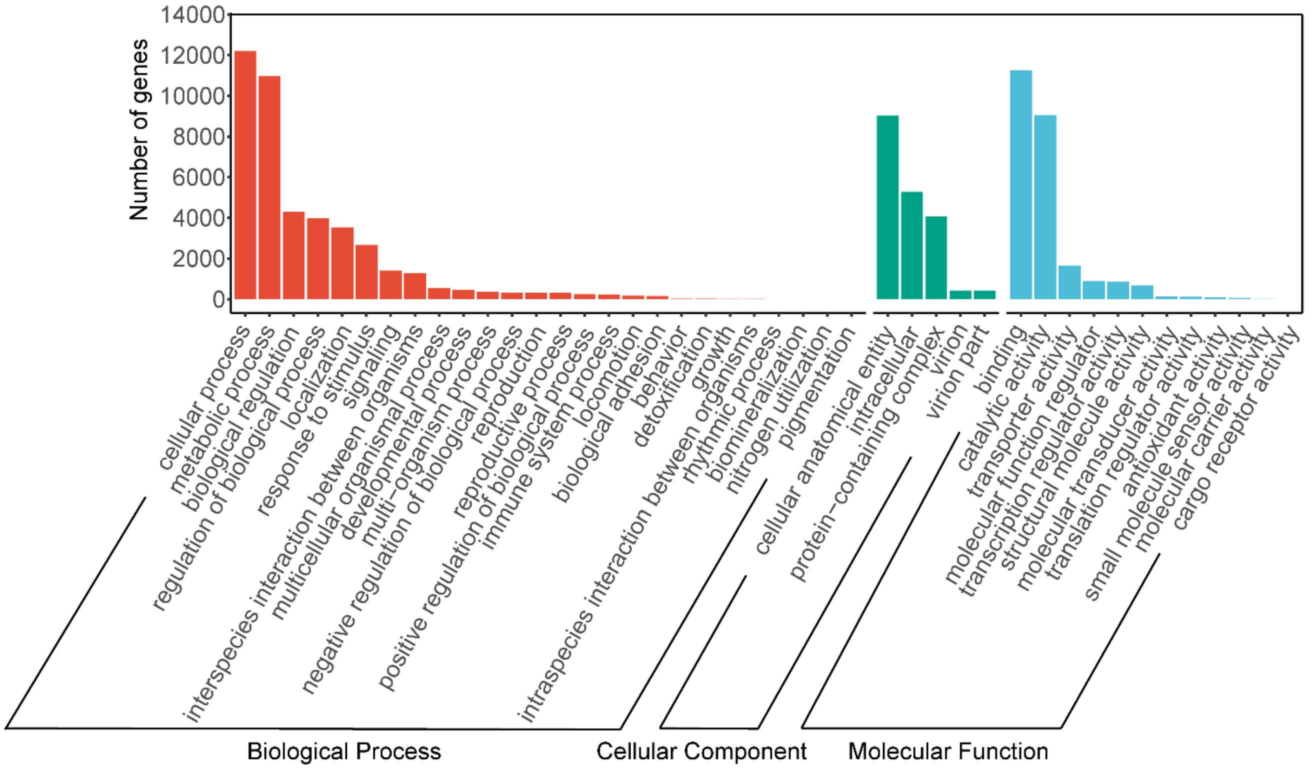

Figure S3. GO function annotation of the unigenes in common vetch seed.

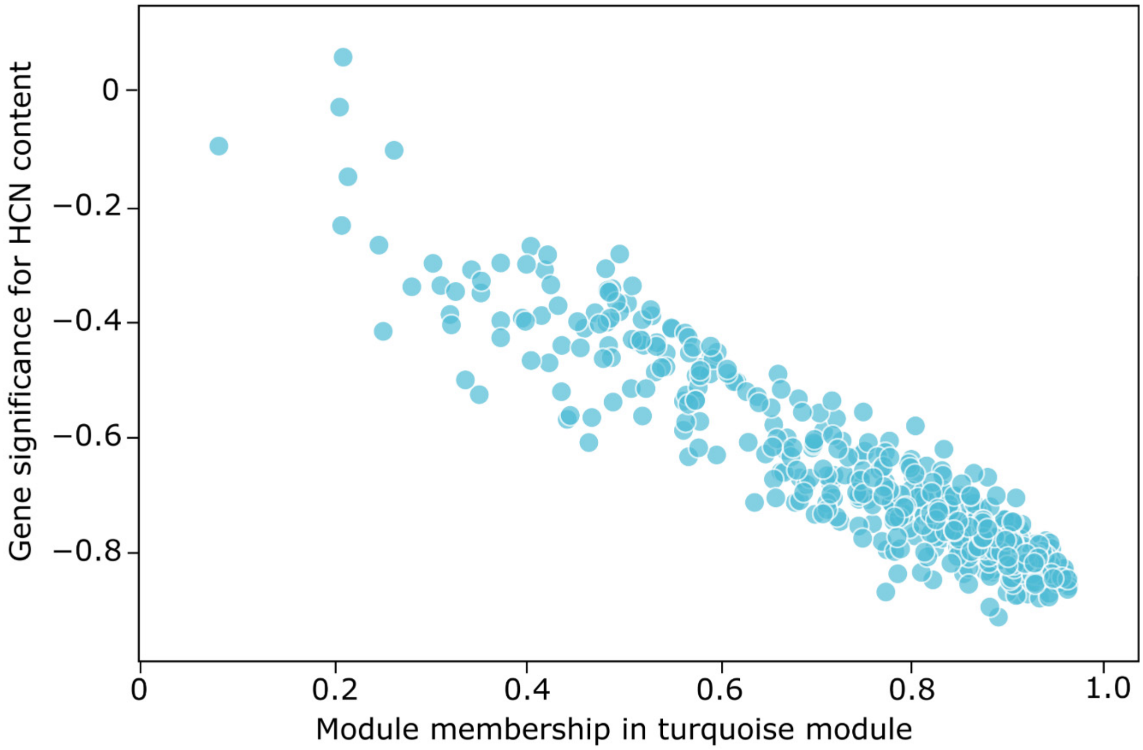

Figure S4. Correlation between module membership (MM) and gene significance (GS) for HCN content in turquoise module.
